# The Effect of the Ketogenic Diet on Adiponectin, Omentin and Vaspin in Children with Drug-Resistant Epilepsy

**DOI:** 10.3390/nu14030479

**Published:** 2022-01-22

**Authors:** Marcin Chyra, Wojciech Roczniak, Elżbieta Świętochowska, Magdalena Dudzińska, Joanna Oświęcimska

**Affiliations:** 1 Department of Paediatric Neurology, Independent Public Healthcare Centre—Municipal Hospital Complex, ul. Władysława Truchana 7, 41-500 Chorzow, Poland; mdudzinskapl@gmail.com; 2 Institute of Medicine, Jan Grodek State University in Sanok, ul. Mickiewicza 21, 38-500 Sanok, Poland; wojciech_roczniak@interia.pl (W.R.); smina@poczta.onet.pl (J.O.); 3 Department of Medical and Molecular Biology, Faculty of Medical Sciences in Zabrze, Medical University of Silesia in Katowice, ul. Jordana 19, 41-808 Zabrze, Poland; elaswieta@interia.pl

**Keywords:** adiponectin, omentin-1, vaspin, ketogenic diet, drug-resistant epilepsy, children

## Abstract

Background: Changes in adipokine secretion may be involved in the anti-epileptic effect of a ketogenic diet (KD) in drug-resistant epilepsy (DRE). Objectives: The assessment of the influence of KD on serum adiponectin, omentin-1, and vaspin in children with DRE. Methods: Anthropometric measurements (weight, height, BMI, and waist-to-hip circumference ratio) were performed in 72 children aged 3–9 years, divided into 3 groups: 24 children with DRE treated with KD, 26—treated with valproic acid (VPA), and a control group of 22 children. Biochemical tests included fasting glucose, insulin, beta-hydroxybutyric acid, lipid profile, aminotransferases activities, and blood gasometry. Serum levels of adiponectin, omentin-1 and vaspin were assayed using commercially available ELISA tests. Results: Serum levels of adiponectin and omentin-1 in the KD group were significantly higher and vaspin—lower in comparison to patients receiving VPA and the control group. In all examined children, serum adiponectin and omentin-1 correlated negatively with WHR and serum triglycerides, insulin, fasting glucose, and HOMA-IR. Vaspin levels correlated negatively with serum triglycerides and positively with body weight, BMI, fasting glucose, insulin, and HOMA-IR. Conclusion: One of the potential mechanisms of KD in children with drug-resistant epilepsy may be a modulation of metabolically beneficial and anti-inflammatory adipokine levels.

## 1. Introduction

The ketogenic diet (KD) is a nonpharmacological metabolic therapy used to treat drug-resistant epilepsy (DRE) in children [1]. The KD strategy involves switching a patient’s metabolism to a state similar to ketosis, which occurs during chronic starvation. This metabolic therapy requires careful dietary planning with balanced macronutrient proportions that result in physiological alterations (i.e., ketosis generation). In children, the KD essentially involves age-appropriate protein content, a high proportion of fat, and reduced carbohydrate intake. Currently, the target lipid to nonlipid ratio (the ketogenic ratio) is most often 4:1 but this depends on the age of the child (3:1 in children under 2 years old). In the KD, around 90% of the daily energy requirement is provided by fats. Ketone bodies (e.g., acetone, acetoacetate, beta-hydroxybutyric acid) are produced from the beta-oxidation of free fatty acids. These are incorporated into the Krebs cycle and thus become a direct source of energy for cells. In comparison, in a conventional diet, as much as 49% of the energy comes from carbohydrates and only 35% from fat [2,3,4].

A major shift in nutrient proportions during KD modifies endocrine activity in adipose tissue by changing the profile of secreted adipokines [5,6]. Since most of these hormones can affect the course of inflammatory processes, this aspect of their activity seems particularly interesting in the context of hypotheses regarding the role of inflammatory processes in the development of epilepsy and its complications [7]. Many studies have demonstrated that numerous substances secreted in inflammatory states of the central nervous system (CNS) or peripheral tissues induce changes in the expression and function of neurotransmitter receptors, leading to a decreased seizure threshold. In the long term, recurrent epileptic seizures damage neurons and the blood–brain barrier, which promotes further activation and propagation of inflammatory processes. By increasing the secretion of pro-inflammatory cytokines, low-grade inflammation can escalate the frequency of seizures and contribute to their generalization, as well as the development of drug resistance and epilepsy complications [8].

Among the vast majority of adipokines, adiponectin, omentin-1, and vaspin have unique, beneficial anti-inflammatory and metabolic effects [9,10,11]. A few reports also point to a potential role of adiponectin deficiency in the process of epileptogenesis; however, the role of omentin-1 and vaspin has not been established, yet [12,13].

Adiponectin is a protein with 224 amino acids produced by adipocytes residing in both white and brown adipose tissue [9,14,15]. Oligomerization facilitates the formation of a low molecular weight complex (LMW) composed of 12–18 polymer molecules in the circulatory system where it can undergo further polymerization into hexamer forms (middle molecular weight complex—MMW) or a high molecular weight complex (HMW), and the proteolytic cleavage of full-length adiponectin results in the formation of the globular form [9]. The biological role of the individual forms has not been clearly defined, however, LMW complexes have potent anti-inflammatory bioactivity while the polymeric forms (HMW) are associated with metabolic processes, strongly interacting in the regulation of tissue sensitivity to insulin [16,17,18]. Omentin-1 protein, secreted by stromal cells of the visceral adipose tissue and endothelial cells, is a 313 amino acid peptide chain containing a signal sequence and a fibrinogen-associated region [19,20]. Vaspin is a serpin from the serine protease inhibitor family, highly expressed in visceral adipose tissue (VAT), but also in different degrees in tissues such as the human stomach, pancreas, liver, and hypothalamus [21].

The most important metabolic pathway regulated by adiponectin, omentin, and vaspin is the adenylate-activated protein kinase (AMPK) cascade that is a major regulator of lipid metabolism and glucose metabolism involved also in the regulation of anti-inflammatory pathways [9,22,23,24,25,26,27]. This kinase is activated by a response to stress factors that deplete the supply of cellular ATP, including hypoglycemia, hypoxia, ischemia, and heat shock [27]. As a cellular energy receptor, AMPK responds to low levels of ATP by inhibiting ATP-consuming biosynthesis processes, including gluconeogenesis, lipid, and protein synthesis. It can also suppress the activation of the nuclear factor-κB (NF-κB) system, a key regulator of innate immunity and inflammation, via several pathways [28]. Interestingly, AMPK is involved in mediating the physiological changes of KD as a component of the AMPK–peroxisome proliferator-activated receptor γ coactivator 1α (PGC-1α) signaling pathway [29]. The beneficial effects of the aforementioned adipokines on carbohydrate metabolism also include an increase in the insulin sensitivity of tissues [9,20,30,31,32]. Adiponectin also stimulates insulin secretion from the pancreas, and vaspin alleviates the native human insulin degradation through the inhibition of a protease—kallikrein 7 [20,30,31,32,33]. The metabolically beneficial effect of adiponectin, omentin-1, and vaspin has been confirmed in human studies. Decreased adiponectin and omentin-1 concentrations have been found in various conditions, such as type 2 diabetes, obesity, and metabolic syndrome [9,15,34,35]. On the other hand, elevated vaspin levels may constitute a compensatory mechanism that occurs in reaction to increased obesity and insulin resistance [36,37].

Adiponectin, omentin-1, and vaspin are considered anti-inflammatory adipokines. It has been demonstrated that they could suppress cytokine-induced NF-κB activation [15,26,34,38]. Adiponectin and omentin-1 can modulate the activity of macrophages, inhibiting the TNF-α production of pro-inflammatory cytokines [15,34,39,40,41]. Moreover, omentin-1 affects many intracellular signaling cascades by inhibiting the inflammatory cyclooxygenase-2 (COX-2) [42]. Vaspin administration in mice resulted in the suppression of pro-inflammatory adipokines: TNF-α, resistin and leptin, while upregulating the levels of adiponectin [33].

The KD is a clinically effective treatment for refractory epilepsy in children, but there is more to explore regarding its mechanism of action and potential new therapeutic approaches associated with the function of adipokines and substances modulating their secretion/activity. This compelled us to study the serum concentrations of selected metabolically beneficial, anti-inflammatory adipokines (adiponectin, omentin-1, and vaspin) in children with drug-resistant epilepsy (DRE) treated with the KD. We compared the results with those obtained from children receiving valproates (VPA) and a control group (children diagnosed with headaches) and analyzed the correlations with anthropometric and metabolic parameters.

## 2. Materials and Methods

The cross-sectional study involved 72 patients from the Department of Paediatric Neurology at the Independent Public Healthcare Centre—Municipal Hospital Complex in Chorzów. There were 40 girls and 32 boys aged 3–9 years without signs of puberty (at Tanner stage I). The participants were divided into 3 groups. The study group (KD) consisted of 24 patients (11 girls, 13 boys) aged 3–9 years (average age 6.2 ± 2.2 years) who were diagnosed with drug-resistant epilepsy and treated for >3 months with the KD. In this period, no modifications in pharmacotherapy were introduced. The exclusion criteria were: known chronic concomitant disease, taking medications (apart from antiepileptic drugs) and/or dietary supplements within the past three months, infections within the last month before the study, and non-compliance with medical recommendations. Before the treatment was started, generalized seizures were observed in 14 children from this group (6 girls and 8 boys). There were polymorphic seizures in 9 of them (5 girls, 4 boys), and 1 boy experienced focal seizures. The KD was the only treatment administered to 3 patients, while 4 children on the KD also received 1 medication (of these, 3 patients received VPA, and 1 received levetiracetam). There were 14 patients on the KD who also received 2 antiepileptic drugs (10 patients received VPA in combination with topiramate, levetiracetam, ethosuximide, or clonazepam, while 4 patients were given levetiracetam in combination with clonazepam or vigabatrin). In the 3 remaining patients, triple pharmacotherapy was used in addition to the KD (with the use of VPA in each case).

Due to the adjuvant nature of KD therapy in children with DRE, medication is unavoidable. As AEDs may be a potential factor influencing the serum adipokines’ levels and the most frequently used drug was VPA; we decided to compare the results obtained in the KD group with children with epilepsy treated with VPA as a monotherapy. This group included 26 patients (16 girls, 10 boys) aged 3–9 years (average age 6.7 ± 2.3 years). The exclusion criteria were: known chronic concomitant disease, taking medications (apart from antiepileptic drugs) and/or dietary supplements within the past three months, infections within the last month before the study, and non-compliance with medical recommendations. In this group, before the treatment was started, generalized seizures were observed in 17 children (10 girls, 7 boys), while 9 (5 girls, 4 boys) presented with focal seizures. The antiepileptic treatment prior to biochemical assays lasted at least 3 months).

The control group (C) was composed of 22 patients (13 girls, 9 boys) aged 3–9 years (average age 7.7 ± 1.6 years) who were admitted to the hospital to determine the cause of headaches. We were unable to collect blood samples from completely healthy children for ethical reasons. The exclusion criteria were: abnormal results of standard laboratory and imaging tests, known chronic concomitant disease, taking medications and/or dietary supplements within the past three months, and infections within the last month before the study.

For each patient, a detailed medical history was obtained, and a meticulous physical examination was performed, including anthropometric measurements (weight, height, waist and hip circumferences, and sexual maturity according to the Tanner scale [43]). The results of the measurements were used to calculate the body mass index (BMI) according to the formula BMI = body weight (kg)/height (m^2^). The waist/hip ratio (WHR) was calculated according to the formula WHR = waist circumference (cm)/hip circumference (cm). Anthropometric measurements were also expressed as standard deviations from the mean values (standard deviation score, SDS) for age and sex. All parameters were matched against percentile growth charts from the OLAF PL0080 research project [44,45].

The number of seizure episodes was assessed based on patient’s diaries.

Venous blood samples of 10 mL were drawn in the morning between 8 AM and 10 AM in the fasted state at the same time as routine laboratory tests during hospitalization. Levels of high-sensitivity C-reactive protein (CRP), fasting blood glucose, beta-hydroxybutyrate (BHBA), alanine aminotransferase (ALT) and aspartate aminotransferase (AST) activities, lipid profile, and gasometry were determined at the Laboratory of the Municipal Hospital Complex in Chorzów. Hormonal tests were performed at the Chair and Department of Medical and Molecular Biology, Faculty of Medical Sciences in Zabrze, Medical University of Silesia in Katowice. Adiponectin, omentin-1, and vaspin concentrations were determined via the enzyme-linked immunosorbent assay (ELISA) method using commercial tests (Bio-Vendor LLC Laboratorní Medicína a.s., Prague, Czech Republic). For insulin levels, the same method was applied using a Mercordia Ultrasensitive Insulin ELISA kit (Mercordia AB, Uppsala, Sweden). The HOMA-IR (homeostasis assessment model—insulin resistance) score was calculated according to the formula:

HOMA-IR = [fasting insulin (μIU/mL) × fasting glucose (mmol/L)]/22.5

The research was approved by the Bioethics Committee of the Medical University of Silesia in Katowice (Approval no. KNW/0022/KB1/155/15 of 15 December 2015). Additionally, we received written consent from the patients’ parents or legal guardians.

### Statistical Analysis

A database was created in a Microsoft Excel spreadsheet. Statistical calculations were done using the software Statistica 10.0 (StatSoft Inc., Tulsa, OK, USA). The Kolmogorov–Smirnov test was used to check whether the tested parameters had a normal distribution. The homogeneity of variances was assessed with Levene’s test. For variables with a normal distribution, group comparisons were performed using a one-way analysis of variance (one-way ANOVA) and Tukey’s HSD post hoc tests. The Kruskal–Wallis test was used if the distribution of variables differed significantly from a normal distribution and Levene’s test showed no homogeneity of variances.

For variables with a normal distribution, linear correlations were established by determining Pearson coefficients. In the case of variables with a distribution significantly deviating from a normal distribution, Spearman coefficients were used. A significance level of α < 0.05 was adopted in statistical calculations. Values in the tables and text are presented as the mean ± SD (minimum–maximum).

## 3. Results

**Figure 1:**
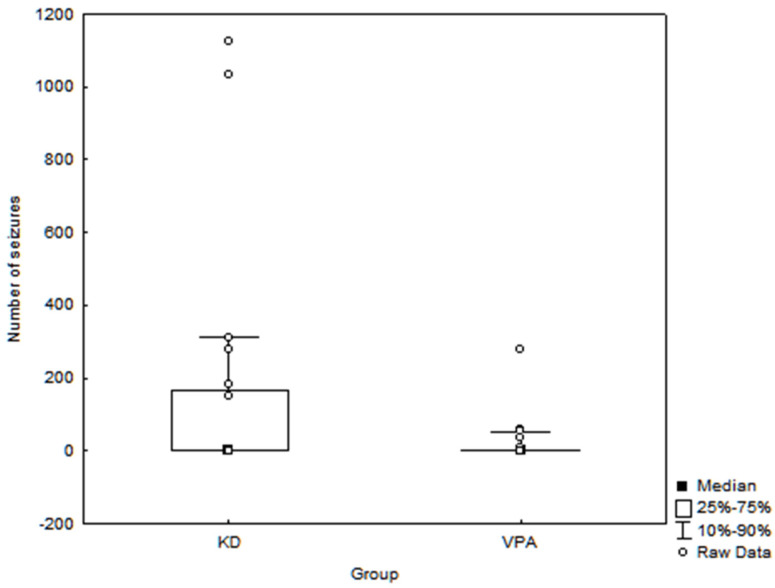
Number of seizure episodes in examined children treated with ketogenic diet (KD) and valproate (VPA).

In the month prior, the blood sampling seizures were observed in 7 children from the KD group and 10 children from the VPA group. In all patients from the KD group who presented seizures, their episodes were multiple during the day, whereas in the VPA group multiple seizure episodes were observed only in one child. The median number of seizure episodes and the interquartile range are presented in Figure 1. 

All examined children were prepubertal (Tanner I). The results of the anthropometric measurements and biochemical assays are shown in Table 1.

There were no significant differences between children 3–5 and 6–9 years of age in all examined groups (KD, VPA, and C) according to the biochemical parameters and serum adiponectin, omentin-1, and vaspin concentrations; therefore, these age subgroups were combined for further analysis (Table S1). Moreover, we did not observe any significant differences in the examined adipokines levels when particular subgroups of children treated with KD (i.e., only KD; KD + 1 AED; KD + 2 AEDs; KD + 3 AEDs) were analyzed. Therefore, for further analysis, these subgroups were combined into the one KD group (Figures S1–S3).

**Figure 2:**
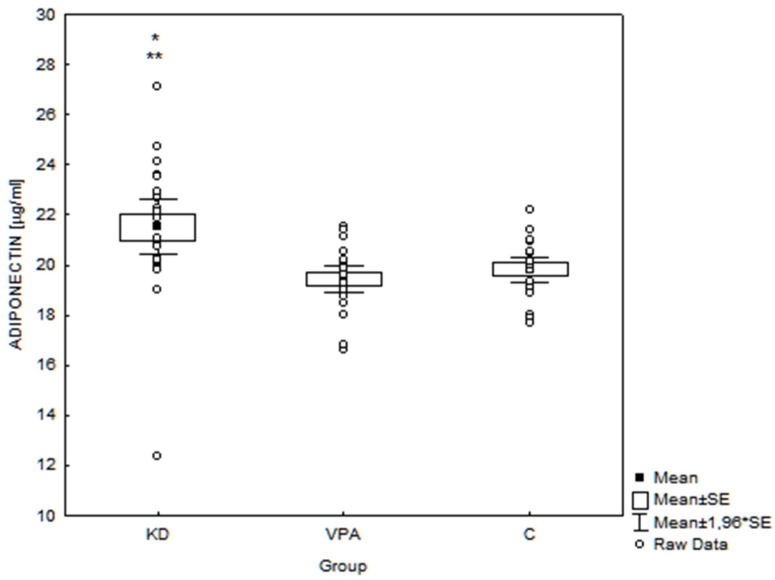
Serum adiponectin concentrations in the examined groups of children (*n* = 72). * *p* = 0.001 KD vs. VPA; ** *p* = 0.005 KD vs. C; SE—standard error; KD—children treated with ketogenic diet group; VPA—children treated with valproate; C—controls.

Adiponectin levels in the KD group (21.50 ± 2.73 μg/mL, range 12.35–27.11 μg/mL) were significantly higher than in the VPA group (19.45 ± 1.29 μg/mL, range 16.61–21.56 μg/mL, *p* = 0.001) and C group (19.80 ± 1.23 μg/mL, range 17.71–22.18 μg/mL, *p* = 0.005) (Figure 2). 

**Figure 3:**
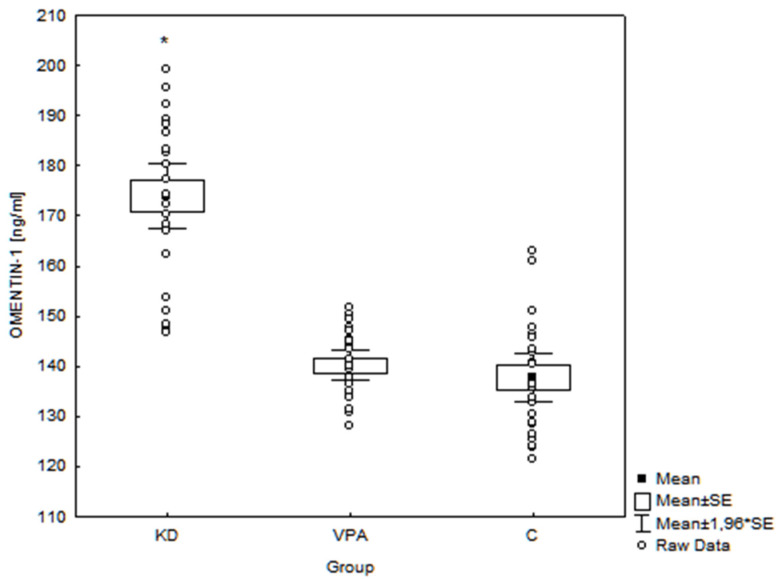
Serum omentin-1 concentrations in the examined groups of children (*n* = 72). * *p* < 0.001 KD vs. VPA and KD vs. C; SE—standard error; KD—children treated with ketogenic diet group; VPA—children treated with valproate; C—controls.

Omentin-1 levels in children treated with the KD (173.95 ± 15.92 ng/mL, range 146.85–199.11 ng/mL) were also significantly higher (*p* = 0.001) than in the VPA group (140.19 ± 7.35 ng/mL, range 128.13–151.69 ng/mL) and C group (137.73 ± 11.65 ng/mL, range 121.48–162.91 ng/mL) (Figure 3). 

**Figure 4:**
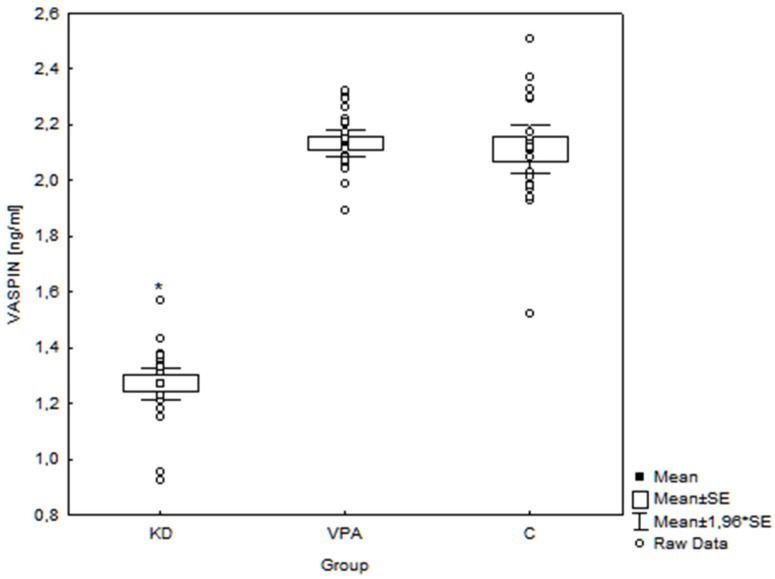
Serum vaspin concentrations in the examined groups of children (*n* = 72). * *p* < 0.001 KD vs. VPA and KD vs. C; SE—standard error; KD—children treated with ketogenic diet group; VPA—children treated with valproate; C—controls.

Conversely, vaspin concentrations in the KD group (1.27 ± 0.14 ng/mL, range 0.92–1.57 ng/mL) were significantly lower (*p* = 0.001) than in the VPA group (2.13 ± 0.12 ng/mL, range 1.89–2.32 ng/mL) and C group (2.11 ± 0.21 ng/mL, range 1.52–2.51 ng/mL) (Figure 4). 

The analysis of correlations did not show any significant relationship between serum adipokines concentrations and the number of seizures, anthropometric, or biochemical parameters in children treated with the KD. We also did not observe any significant correlation between the number of seizures and serum BHBA in this group. Table 2 shows the results of the correlation analysis between anthropometric parameters, biochemical assays, and serum adipokine levels in all examined children.

## 4. Discussion

### 4.1. Ketogenic Diet and Adipokines

The KD is one of the non-pharmacological methods used to treat DRE. However, despite many years of clinical success with around a 20% reduction in neuronal excitability and epileptic seizures, the mechanisms underlying the anticonvulsant action of the KD remain poorly elucidated [4,43,44,45]. Ketone bodies and the metabolic changes linked with reduced glucose oxidation have a multidirectional anticonvulsant and neuroprotective impact due to reduced intracellular ROS and cellular metabolic stress resulting in an increased fuel supply to neurons. The KD also modifies neurotransmission by increasing glutamate to glutamine conversion and gamma-aminobutyric acid (GABA) synthesis, inhibiting glutamate-mediated hyperexcitability as well as raising tissue noradrenaline levels that may modulate the propensity to develop seizures. The anticonvulsant action of the KD also depends on the activation of ATP-sensitive potassium channels via the increased activity of the Na+/K+ pump, adenosine accumulation, reduction in tyrosine kinase B, epigenetic modifications, and the modulation of hormonal processes and the immune response [4,44,45,46,47]. Several peripheral peptide hormones involved in the control of food intake and metabolism (e.g., leptin, adiponectin, NPY, insulin, ghrelin) that are influenced by the KD also possess antiseizure properties and, as such, are promising candidates for future research on new treatments for epilepsy [45]. 

Adipokines link changes in body metabolism with central nervous system functions and the modulation of inflammatory processes. Reports published over the past two decades provide a growing body of evidence regarding the role played by a dysfunctional immune system and inflammation during epileptogenesis [48,49,50]. It seems that the brain is not a privileged organ in terms of immunity, despite the existence of the blood–brain barrier and chronic inflammatory conditions resulting from glial dysfunction, which may contribute to the occurrence or aggravation of various central nervous system disorders [51,52,53]. Additionally, peripheral or systemic inflammation may provoke an inflammatory response in the CNS, causing exacerbation of ictal activity due to the modulation of the activity of drug-metabolizing enzymes and systems that transport drugs into the brain [54,55,56].

Given these findings, changes in serum adipokine levels in patients using KD therapy would be extremely interesting considering the links between the low-grade inflammation within the CNS or peripheral tissues and the phenomenon of drug resistance in epilepsy, along with the anti-inflammatory effects of the KD [57]. Our results indicate that one of the potential mechanisms behind the KD’s efficacy in children with DRE may be changes in serum levels of anti-inflammatory, metabolically beneficial adipokines: adiponectin, omentin-1, and vaspin.

In our study, serum adiponectin and omentin-1 levels in children with DRE treated with KD were significantly higher and vaspin levels—lower than in patients treated with VPA and controls. Moreover, we did not observe any significant difference in the concentrations of these adipokines between the latter two groups. Serum adiponectin and omentin-1 correlated positively with BHBA levels, whereas in the case of vaspin this relationship was negative. 

The possible mechanisms of KD’s impact on serum adipokines concentrations may include metabolic and anti-inflammatory effects as well as epigenetic changes. 

Recently, it has been postulated that the increased levels of an essential coenzyme that regulates redox metabolism—the oxidized form of nicotinamide adenine dinucleotide (NAD+) and thus an altered ratio of NAD+/NADH—may be a potential fundamental starting point for multiple mechanisms proposed to underly KD action [58]. Indeed, a study on adipocyte-specific nicotinamide phosphoribosyltransferase (Nampt) knockout (ANKO) mice, which showed markedly decreased NAD+ concentrations in WAT, demonstrated the suppression of adiponectin activation and peroxisome proliferator-activated receptor (PPAR) signaling pathways [59]. The latter mechanism may directly stimulate the secretion of adiponectin and omentin-1 through an increase in the number of small adipocytes [16,60]. PPARs are also involved in the control of genes involved in anti-inflammatory and anti-oxidant pathways, which in turn may regulate adipokines secretion via reduced TNF-α and IL-6 levels [61,62]. Another potential, but not tested hypothesis explaining the effect of KD on examined adipokines secretion may be adenosine-dependent epigenetic changes [63] as well as epigenetic mechanisms mediated by the alteration of the gut microbiota [64,65].

The lack of correlation between examined adipokines and the number of seizures either in the KD group or all examined children with epilepsy does not exclude the potential participation of adipokines in the anticonvulsant effect of KD. While the efficacy of the KD in the clinical arena is clearly established, clinical and a few experimental studies have shown that blood ketone levels do not correlate directly with seizure control [66]. This suggests that mechanisms underlying the anti-epileptic effect of KD are more complex and may be mediated also through the modulation of adipokine secretion. The correlations between BHBA and adiponectin, omentin-1, and vaspin observed by us support this hypothesis.

#### 4.1.1. Adiponectin

Adiponectin may confer an anticonvulsant role by protecting the integrity of vascular endothelial cells. In animal models, this adipokine retains the integrity of the blood–brain barrier (BBB) and reduces neuronal cell loss [13]. Adiponectin knock-out mice exhibit increased kainic acid-induced seizure severity, correlated with metabolic disturbances (glucose intolerance, hyperlipdemia, free fatty acids, and increased fat mass) as well as increased hippocampal pathology [12]. Further research found that the protective effect of adiponectin on vascular endothelial cells may be related to its anti-inflammatory properties [67,68]. In a study carried out in mice, Tang et al. [69] indicated adiponectin levels decreased in environments with increased oxidative stress.

Human studies revealed that serum adiponectin levels are significantly lower in patients with epilepsy than among healthy controls [70,71]. Interestingly, serum/plasma adiponectin levels are elevated in children with febrile seizures and in adults 24 h after a seizure episode [72,73]. These findings suggest that elevated adiponectin at least partially released during primary or secondary generalized tonic-clonic seizures from skeletal muscle may play an anti-inflammatory role, thereby reducing brain damage caused by seizures. The potential mechanism underlying this neuroprotection may be related to the regulation of BBB integrity [73].

A similar but longitudinal effect may be achieved through the KD. However, data on serum adiponectin levels in subjects on the KD are scarce and inconsistent. To our knowledge, only three studies performed on children have been published to date [74,75,76]. There were not any significant differences in serum adiponectin levels in children with glucose transporter 1 deficiency syndrome (GLUT1 DS) treated with the KD and antiepileptic drugs (AEDs) [74] or only KD [75]. These findings may arise from the small size of the groups, wide range of age of examined children, or too short observation period. The authors of the above-mentioned studies did not provide any data on BHBA concentrations in their patients, which is relevant considering that BHBA induces adiponectin secretion in adipocytes through the GPR109A receptor [29].

Another pediatric study showed a significant increase in the concentration of HMW adiponectin in obese teenagers on KD without caloric restriction and the hypocaloric diet for 6 months [76]. The same findings have been demonstrated in obese men during the switch from baseline to an isocaloric KD [77] and in healthy young adults switched to a very low-carbohydrate, high-fat (VLCHF) diet without calorie restriction [78]. In addition, a significant increase in adiponectin to leptin ratio (Adpn/Lep) has been reported [78].

Increases in plasma adiponectin during the KD would presumably promote insulin sensitivity [77]. Our results support this hypothesis. Namely, we did not observe any significant relationships between serum adiponectin levels and weight or BMI. Conversely, serum adiponectin levels correlated negatively with insulin, HOMA-IR, and WHR—parameters more related to insulin resistance/insulin sensitivity than BMI or weight [79]. We also observed a significant positive relationship between adiponectin and BHBA in line with Sherrier and Li [29], who suggest that the level of nutritional ketosis is an important determinant of the extent to which the KD influences AMPK activity through adiponectin.

Considering the influence of KD on adiponectin levels in patients with epilepsy, one must be aware of the medication effects. In most studies, treatment with VPA results in hypoadectinemia associated with weight gain and insulin resistance [80,81,82,83,84,85]. The exact pathogenic mechanisms of hypoadectinemia in patients taking VPA remain unclear, but it is most likely multifactorial and may include direct interaction between VPA and adiponectin expression as well as changes in insulin resistance, weight gain, and fat tissue distribution [81,86]. In vitro animal studies have demonstrated that VPA inhibits adiponectin gene expression in “a dose and time-dependent” manner [86]. However, there is no published data regarding such an effect in humans.

Few studies have shown that low adiponectin levels are involved in VPA-induced insulin resistance independent of the effect of excess adiposity [81,83,87]. This drug may also have a direct effect on ß-cell regulation and insulin secretion or interfere with insulin metabolism in the liver, resulting in higher insulin concentrations in the peripheral circulation. Other hypotheses for the mechanism underlying VPA-induced insulin resistance include increased plasma-free fatty acid levels, ß-cell dysfunction secondary to oxidative stress, and an alteration of the sympathetic nervous system via hypothalamic neurons [83].

Contrary to the above-mentioned observations, Sonmez et al. [88] reported that serum adiponectin levels in prepubertal children with epilepsy treated with VPA for 12 months were similar to their pre-treatment values. It should be noted that none of their patients became obese during the observation period. These results are in line with our findings as we did not observe any significant differences in serum adiponectin concentrations between children treated with VPA and healthy controls. However, in our study, weight, BMI, waist circumference, WHR, serum insulin, and HOMA-IR were similar in the VPA and control groups. Conversely, children treated with the KD, despite concomitant treatment with VPA, had higher adiponectin levels, suggesting that the KD may mitigate the adverse metabolic and pro-inflammatory effects of VPA by increasing adiponectin levels and enhancing insulin sensitivity [87].

#### 4.1.2. Omentin-1

The available literature does not contain any data on serum omentin-1 concentration either during KD or in patients with epilepsy. Some data suggest that omentin-1 may confer beneficial metabolic, anti-inflammatory, and neuroprotective effects. It probably increases the susceptibility of adipose tissue to insulin and, therefore, a reduced omentin level can lead to insulin resistance. In patients with type 2 diabetes and obese people, omentin levels are decreased [89]. Conversely, in another human model of chronic starvation, anorexia nervosa serum omentin-1 levels were decreased [90]. Recent studies suggest that omentin-1 plays a neuroprotective role through direct or indirect anti-inflammatory actions, the inhibition of oxidative stress in mitochondria, and the regulation of endothelial function [91].

#### 4.1.3. Vaspin

Serum vaspin concentrations were evaluated by Meral et al. [82] in a group of 44 children with idiopathic, generalized epilepsy treated with VPA compared with healthy controls. Similarly, they did not observe any significant differences between the examined groups; however, their patients had higher BMIs and HOMA-IR, than the control group. To the best of our knowledge, no studies on serum vaspin levels in subjects on the KD have been published yet and the results obtained using different models of chronic starvation are divergent [92,93,94,95]. The contradictory changes in vaspin levels may be related to targeted mechanisms of action. If the intervention or pathology directly increases its production, this will increase the activity of this adipokine and improve metabolic parameters (vaspin-mediated compensation). Conversely, conditions that improve metabolic parameters without invoking vaspin activity would decrease its levels because it is no longer needed (decompensation) [96]. According to this hypothesis, decreased vaspin levels in children treated with the KD may be the consequence of increased insulin sensitivity rather than its causative agent.

### 4.2. Study Limitations and Advantages

There are a few limitations to this study. Firstly, it is a cross-sectional study, and the groups were relatively small. In most cases, children from the KD group also received additional pharmacotherapy. However, for clinical reasons, standardization of the KD group in terms of the anti-epileptic drugs administered was not feasible. In our research, we evaluated serum concentrations of omentin-1 and vaspin in prepubertal children with DRE treated with the KD for the first time. Serum adiponectin levels were assessed only in small groups of children with GLUT1 DS epilepsy. The primary advantage of our study is the comparison of the results obtained in children with DRE treated with the KD to those of not only healthy children but also patients with epilepsy receiving VPA. Another merit of this study is its extensive assessment of metabolic parameters, which cover carbohydrate metabolism, lipid metabolism, and acid–base balance.

## 5. Conclusions

Our results demonstrated that serum adiponectin and omentin-1 levels were significantly higher and vaspin was lower in children with DRE treated with the KD than in patients treated with VPA alone and healthy controls. Therefore, we suggest that one of the potential mechanisms of the KD in children with DRE may be the modulation of metabolically beneficial, anti-inflammatory adipokine levels.

## Figures and Tables

**Table 1:** Results of anthropometric measurements and biochemical assays performed on the examined children.

Parameter	Group			
	KD (*n* = 24)	VPA (*n* = 26)	C (*n* = 22)	*p*
	Mean ± SD (min–max)			
Age [years]	6.2 ± 2.2 (3.0–9.9)	6.7 ± 2.3 (3.1–9.9)	7.7 ± 1.6 (3.6–9.7)	0.84
Body mass [kg]	19.73 ± 7.59 (9.1–39.0)	24.76 ± 9.43 (15.5–54.1)	28.25 ±6.7 (17.0–40.7)	0.08
Height [cm]	112.65 ± 15.82 (86.0–138.0)	120.4 ± 16.41 (95.0–157.5)	129.17 ± 9.83 (103.0–141.5)	0.07
BMI [kg/m^2^]	15.05 ± 2.27 (11.23–21.84)	16.55 ± 2.57 (11.36–21.81)	16.74 ± 2.46 (13.73–23.71)	0.11
Waist circ. [cm]	50.58 ± 7.76 (39.5–67.0)	54.98 ± 6.65 (45.0–74.0)	58.15 ± 6.45 (48.0–74.0)	0.05
Hip circ. [cm]	56.87 ± 10.12 (40.5–79.0)	62.55 ± 8.32 (51.0–89.0)	68.67 ± 8.34 (55.0–84.0)	0.01 *
WHR	0.89 ± 0.05 (0.74–0.98)	0.88 ± 0.05 (0.78–1.00)	0.85 ± 0.06 (0.71–0.98)	0.1
Total cholesterol [mg/dL]	171.6 ± 42.12 (87.0–279.0)	162.02 ± 29.92 (112.0–235.0)	171.07 ± 27.54 (127.0–257.0)	0.81
LDL cholesterol (mg/dL)	102.27 ± 34.34 (38.0–180.6)	96.54 ± 28.03 (58.3–161.3)	101.76 ± 23.51 (54.3–159.6)	0.51
HDL cholesterol [mg/dL]	53.10 ± 17.04 (26.0–102.0)	51.74 ± 8.5 (35.5–65.0)	57.28 ± 12.8 (36.6–88.2)	0.16
Triglycerides [mg/dL]	103.1 ± 53.34 (29.0–302.0)	67.03 ± 43.79 (26.0–279.0)	67.63 ± 37.52 (31.0–204.0)	0.001 * ^#^
AST [IU/L]	30.2 ± 40.61 (6.0–219.0)	27.2 ± 7.37 (18.0–43.0)	26.8 ± 4.57 (19.0–37.0)	0.19
ALT [IU/L]	35.37 ± 22.27 (11.0–120.0)	15.43 ± 8.8 (6.0–54.0)	15.77 ± 3.86 (9.0–22.0)	0.04 ^#^
Fasting glucose [mg/dL]	72.03 ± 8.98 (53.0–90.0)	86.47 ± 7.94 (64.0–101.0)	88.5 ± 7.05 (74.0–101.0)	0.001 * ^#^
Insulin [μU/mL]	2.48 ± 0.64 (1.34–3.75)	4.19 ± 0.64 (3.13–5.3)	4.55 ± 0.92 (3.09–5.98)	0.001 * ^#^
HOMA-IR	0.44 ± 0.13 (0.22–0.7)	0.91 ± 0.18 (0.66–1.29)	1.0 ± 0.23 (0.65–1.51)	0.001 * ^#^
CRP [mg/L]	0.9 ± 1.44 (0.2–5.6)	0.56 ± 0.72 (0.2–3.8)	1.87 ± 3.16 (0.1–5.7)	0.42
HCO^3−^ [mmol/L]	19.95 ± 2.03 (16.5–24.1)	22.62 ± 1.68 (19.5–25.5)	21.95 ± 1.08 (19.1–24.3)	0.001 * ^#^
BE [mEq/L]	−4.39 ± 2.14 (−7.9–0.4)	−1.68 ± 1.75 (−4.9–0.9)	−2.35 ± 1.05 (−4.2–(−0.5))	0.001 * ^#^
BHBA [mmol/L]	4.31 ± 1.62 (1.0–7.3)	0.29 ± 0.23 (0.1–1.3)	0.29 ± 0.25 (0.1–1.3)	0.001 * ^#^

KD—patients with epilepsy treated with the ketogenic diet and pharmacotherapy; VPA—group of patients with epilepsy treated with valproates; C—control group; SD—standard deviation; BMI—body mass index; WHR—waist/hip ratio; SDS—standard deviation score; AST—aspartate aminotransferase; ALT—alanine aminotransferase; HOMA-IR—insulin resistance score; CRP—C-reactive protein; HCO^3−^—bicarbonate level; BE—base excess; BHBA—beta-hydroxybutyrate; * significant differences KD vs. C; ^#^ significant differences KD vs. VPA.

**Table 2:** Analysis of correlation between anthropometric parameters and biochemical assays and the concentrations of tested adipokines in all examined children (*n* = 72).

Parameter	Adiponectin [μg/mL]	Omentin-1 [ng/mL]	Vaspin [ng/mL]
Age (years)	r = −0.04 *p* = 0.72	r = −0.15 *p* = 0.22	r = 0.02 *p* = 0.86
Number of seizures/month ^#^	r = 0.06 *p* = 0.69	r = 0.08 *p* = 0.61	r = −0.08 *p* = 0.60
Body mass (kg)	r = −0.22 *p* = 0.07	r = −0.34 *p* < 0.01 *	r = 0.28 *p* = 0.02*
Height (cm)	r = −0.14 *p* = 0.24	r = −0.31 *p* = 0.01 *	r = 0.19 *p* = 0.11
BMI (kg/m^2^)	r = −0.21 *p* = 0.08	r = −0.22 *p* = 0.07	r = 0.25 *p* = 0.04 *
Waist circ. (cm)	r = −0.19 *p* = 0.11	r = −0.21 *p* = 0.08	r = 0.21 *p* = 0.09
Hip circ. (cm)	r = −0.29 *p* = 0.02 *	r = −0.27 *p* = 0.03 *	r = 0.21 *p* = 0.08
WHR	r = −0.34 *p* = 0.01 *	r = −0.29 *p* = 0.02 *	r = −0.11 *p* = 0.38
Total cholesterol (mg/dL)	r = 0.15 *p* = 0.21	r = 0.14 *p* = 0.25	r = −0.17 *p* = 0.17
LDL cholesterol (mg/dL)	r = 0.11 *p* = 0.38	r = 0.06 *p* = 0.64	r = −0.08 *p* = 0.51
HDL cholesterol (mg/dL)	r = −0.03 *p* = 0.82	r = −0.01 *p* = 0.94	r = −0.04 *p* = 0.75
Triglycerides (mg/dL)	r = −0.36 *p* < 0.01 *	r = −0.39 *p* < 0.01 *	r = −0.36 *p* < 0.01 *
AST (IU/L)	r = 0.26 *p* = 0.03 *	r = 0.05 *p* = 0.69	r = −0.05 *p* = 0.69
ALT (IU/L)	r = 0.31 *p* = 0.01 *	r = 0.12 *p* = 0.32	r = −0.15 *p* = 0.22
Fasting glucose (mg/dL)	r = −0.34 *p* < 0.01 *	r = −0.49 *p* < 0.01 *	r = 0.50 *p* < 0.01 *
Insulin (μU/mL)	r = −0.32 *p* = 0.01 *	r = −0.59 *p* < 0.01 *	r = 0.66 *p* < 0.01 *
HOMA-IR	r = −0.35 *p* < 0.01 *	r = −0.62 *p* < 0.01 *	r = 0.65 *p* < 0.01 *
CRP (mg/L)	r = 0.10 *p* = 0.42	r = 0.05 *p* = 0.72	r = 0.09 *p* = 0.49
HCO^3−^ (mmol/L)	r = −0.26 *p* = 0.03 *	r = −0.41 *p* < 0.01 *	r = 0.41 *p* < 0.01 *
BE (mEq/L)	r = −0.32 *p* = 0.01 *	r = −0.41 *p* < 0.01 *	r = 0.42 *p* < 0.01 *
BHBA (mmol/L)	r = 0.44 *p* < 0.01 *	r = 0.58 *p* < 0.01 *	r = −0.76 *p* < 0.01 *

BMI—body mass index; WHR—waist/hip ratio; SDS—standard deviation score; AST—aspartate aminotransferase; ALT—alanine aminotransferase; HOMA-IR—insulin resistance score; CRP—C-reactive protein; HCO^3−^—bicarbonate level; BE—base excess; BHBA—beta-hydroxybutyrate; * significant correlations; ^#^ calculated for children with epilepsy (*n* = 50).

## Data Availability

The data presented in this study are available on request from the corresponding author.

## Supplementary Materials

The following are available online at https://www.mdpi.com/article/10.3390/nu14030479/s1, Figure S1: Results of serum adiponectin concentrations in the examined children treated with KD according to concomitant medication; Figure S2: Results of serum omentin-1 concentrations in the examined children treated with KD according to concomitant medication; Figure S3: Results of serum vaspin concentrations in the examined children treated with KD according to concomitant medication; Table S1: Results of serum adiponectin, omentin-1 and vaspin concentrations in the examined children aged 3–5 and 6–9 years.
