# Supplementary material for: The Effect of the Ketogenic Diet on Adiponectin, Omentin and Vaspin in Children with Drug-Resistant Epilepsy
Source: Nutrients. 2022 Jan 22;14(3):479. doi: 10.3390/nu14030479 (PMC8839826; doi:10.3390/nu14030479)
Supplement: Supplementary file 1 [file nutrients-14-00479-s001.zip › nutrients-1546464-supplementary.pdf]

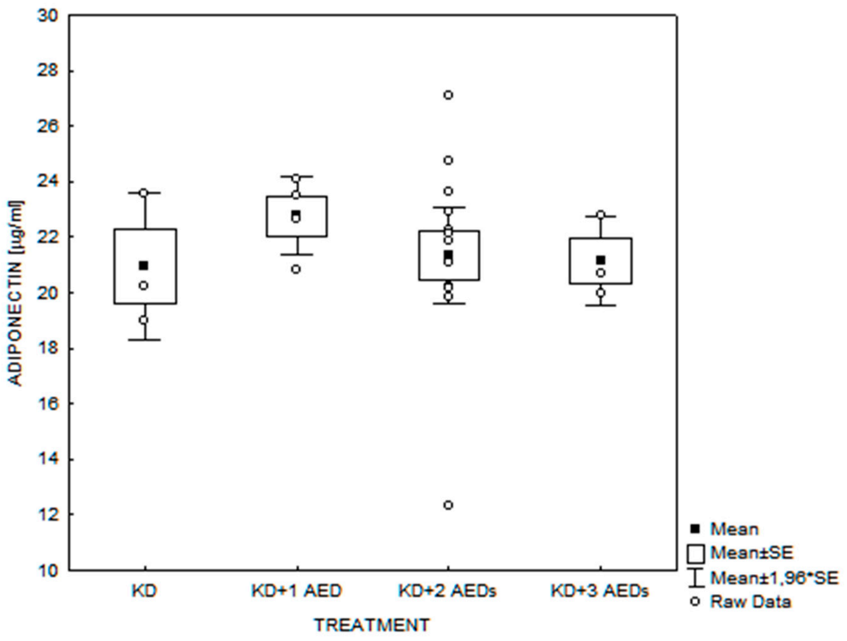

Figure S1. Results of serum adiponectin concentrations in the examined children treated with KD according to concomitant medication

p=0.80 (one-way ANOVA test); KD – ketogenic diet; AED – anti-epileptic drug; SE – standard error

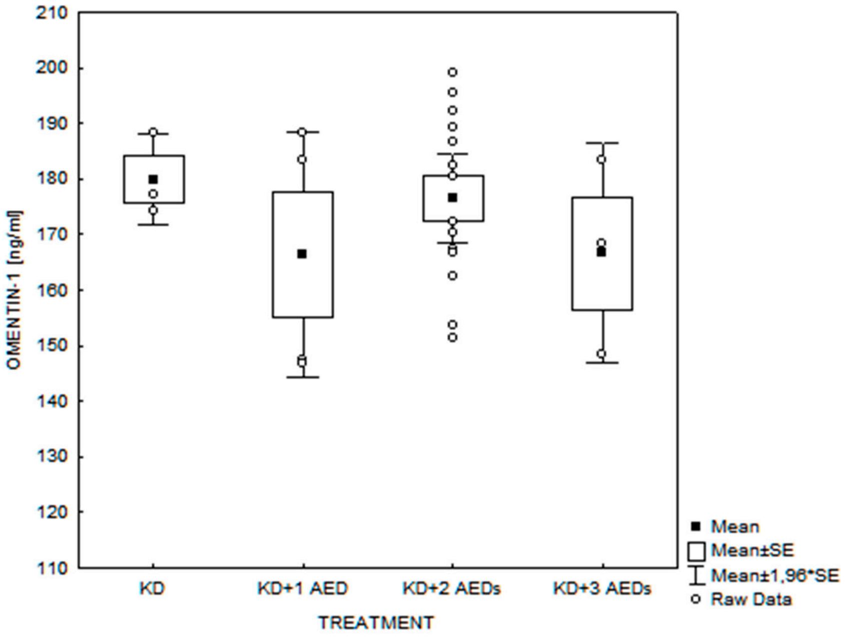

Figure S2. Results of serum omentin-1 concentrations in the examined children treated with KD according to concomitant medication

p=0.54 (one-way ANOVA test); KD – ketogenic diet; AED – anti-epileptic drug; SE – standard error

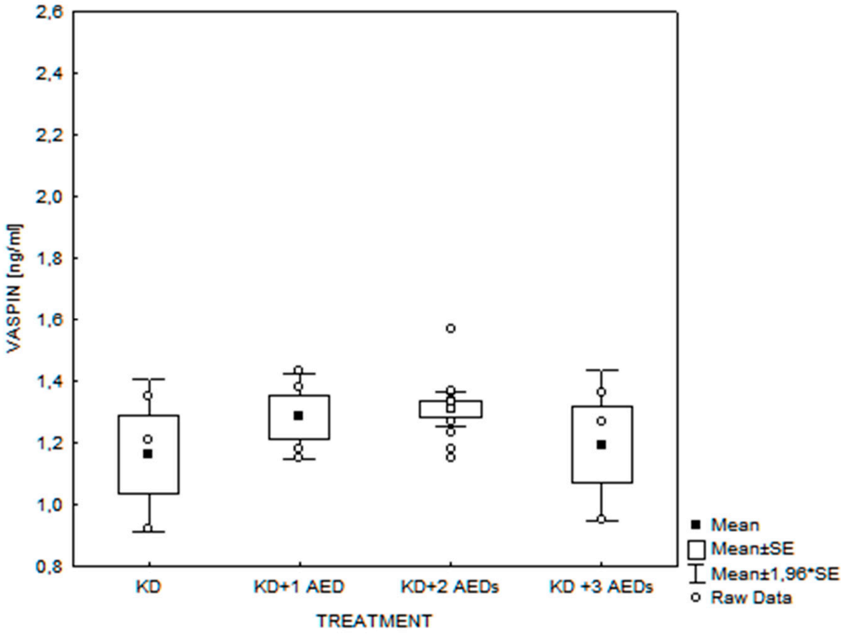

Figure S3. Results of serum vaspin concentrations in the examined children treated with KD according to concomitant medication

p=0.31 (one-way ANOVA test); KD – ketogenic diet; AED – anti-epileptic drug; SE – standard error

Table S1. Results of serum adiponectin, omentin-1 and vaspin concentrations in the examined children aged 3-5 and 6-9 years

**Parameter**	**Group**								
	**KD (n=24)**			**VPA (n=26)**			C (n=22)		
**age [years]**	**3-5 (n=4)**	**6-9 (n=20)**	**p**	**3-5 (n=7)**	**6-9 (n=19)**	p	3-5 (n=4)	6-9 (n=18)	p
	mean ± SD (range)			mean ± SD (range)			mean ± SD (range)		
adiponectin [µg/ml]	21.62 ± 1.73 (20.15-23.53)	21.47 ± 2.93 (12.35-27.11)	0.92	19.31 ± 1.58 (16.61- 21.40)	19.50 ± 1.22 (16.82- 21.56)	0.39	19.55 ± 1.39 (17.88-20.91)	19.87 ± 1.22 (17,71-22.18)	0.65
omentin-1 [ng/ml]	168.38 ± 25.78 (146.85-199.11)	175.06 ±13.93 (148.38-195.42)	0.46	141.11 ± 8.66 (130.67-151.69)	139.87 ± 7.10 (128.13-150.41)	0.49	142.56 ± 16.42 (125.43-161.10)	136,66 ± 10,65 (121.48-162.91	0.37
vaspin [ng/ml]	1.25 ± 0.10 (1.15-1.33)	1.28 ± 0.15 (0.92-1,57)	0.72	2.13 ± 0.16 (1.89-2.31)	2.13 ± 0.10 (1.89-2.32)	0.15	2.24 ± 0.14 (2.08-2.37)	2.07 ± 0.21 (1.52-2.51)	0.19

KD – patients with epilepsy treated with the ketogenic diet and pharmacotherapy; VPA – group of patients with epilepsy treated with valproates; C – control group; SD – standard deviation
